# Common variants at the 9q22.33, 14q13.3 and ATM loci, and risk of differentiated thyroid cancer in the Cuban population

**DOI:** 10.1186/s12863-015-0180-5

**Published:** 2015-03-01

**Authors:** Celia M Pereda, Fabienne Lesueur, Maroulio Pertesi, Nivonirina Robinot, Juan J Lence-Anta, Silvia Turcios, Milagros Velasco, Mae Chappe, Idalmis Infante, Marlene Bustillo, Anabel García, Enora Clero, Constance Xhaard, Yan Ren, Stéphane Maillard, Francesca Damiola, Carole Rubino, Sirced Salazar, Regla Rodriguez, Rosa M Ortiz, Florent de Vathaire

**Affiliations:** Institute of Oncology and Radiobiology, Havana, Cuba; The French National Institute of Health and Medical Research (Inserm), U900, Institut Curie, Mines ParisTech, Paris, F-75005 France; Genetic Cancer Susceptibility, International Agency for Research on Cancer (IARC), Lyon, F-69372 France; National Institute of Endocrinology, Havana, Cuba; Cuban Health Public Ministry, Havana, Cuba; The French National Institute of Health and Medical Research (Inserm), Centre for Research in Epidemiology and Population Health (CESP), U1018, Radiation Epidemiology Group, Villejuif, 94805 France; Paris-Sud University, Villejuif, 94805 France; Institut Gustave Roussy (IGR), Villejuif, 94805 France; CRCL, CNRS UMR5286, the French National Institute of Health and Medical Research (Inserm) U1052, Centre Léon Bérard, Lyon, F-69008 France

**Keywords:** Differentiated thyroid carcinoma, Cuba, Genetic susceptibility, ATM, FOXE1, NKX2-1, Polymorphism

## Abstract

**Background:**

The incidence of differentiated thyroid carcinoma (DTC) in Cuba is low and the contribution of host genetic factors to DTC in this population has not been investigated so far. Our goal was to assess the role of known risk polymorphisms in DTC cases living in Havana. We genotyped five polymorphisms located at the DTC susceptibility loci on chromosome 14q13.3 near *NK2 homeobox 1 (NKX2-1)*, on chromosome 9q22.33 near *Forkhead factor E1 (FOXE1)* and within the DNA repair gene *Ataxia-Telangiectasia Mutated (ATM)* in 203 cases and 212 age- and sex- matched controls. Potential interactions between these polymorphisms and other DTC risk factors such as body surface area, body mass index, size, ethnicity, and, for women, the parity were also examined.

**Results:**

Significant association with DTC risk was found for rs944289 near *NKX2-*1 (OR _per A allele_ = 1.6, 95% CI: 1.2–2.1), and three polymorphisms near or within *FOXE1*, namely rs965513 (OR _per A allele_ = 1.7, 95% CI: 1.2–2.3), rs1867277 in the promoter region of the gene (OR _per A allele_ = 1.5, 95% CI: 1.1–1.9) and the poly-alanine tract expansion polymorphism rs71369530 (OR _per Long Allele_ = 1.8, 95% CI: 1.3–2.5), only the 2 latter remaining significant when correcting for multiple tests. Overall, no association between DTC and the coding SNP D1853N (rs1801516) in *ATM* (OR _per A Allele_ = 1.1, 95% CI: 0.7–1.7) was seen. Nevertheless women who had 2 or more pregnancies had a 3.5-fold increase in risk of DTC if they carried the A allele (OR 3.5, 95% CI: 3.2–9.8) as compared to 0.8 (OR 0.8, 95% CI: 0.4–1.6) in those who had fewer than 2.

**Conclusions:**

We confirmed in the Cuban population the role of the loci previously associated with DTC susceptibility in European and Japanese populations through genome-wide association studies. Our results on *ATM* and the number of pregnancies raise interesting questions on the mechanisms by which oestrogens, or other hormones, alter the DNA damage response and DNA repair through the regulation of key effector proteins such as *ATM*. Due to the small size of our study and to multiple tests, all these results warrant further investigation.

## Background

Cuba is the largest island in the Caribbean Sea, with an estimated population of over 11 million people according to the 2012 census [1]. The Cuban population has a mixed ethnic composition, with a large proportion of people of African or Spanish origin. Before the Spanish colonisation, Cuba was occupied by Native Americans who had migrated from the mainland of North, Central and South America several centuries before [2]. Today, Afro-Cubans, mostly from Congo, account for 35% of the population [1]. The registered incidence of DTC is low in Cuba, being 4.1 per 100,000 in females and 1.0 in males [1,3]*.*

In a previous case-control study on thyroid cancer risk factors conducted in Cuba [4], we showed that DTC risk was lower in populations of African origin, was increased with parity and body surface area, and was higher in farmers than in people pursuing other types of activities. A history of ionizing radiation, agricultural occupation and an artesian well as the main source of drinking water were also associated with a significantly increased risk of developing DTC. In women, irregular cycles and menopause status were associated with a higher risk of DTC. On the other hand, DTC risk was lower in current or former smokers than in non-smokers [4].

We investigated the The contribution of host genetic factors to DTC susceptibility has never been assessed in the Cuban population. Since *NKX2-1* (*NK2 homeobox 1*, also called *TTF1* for *Thyroid Transcription Factor 1*), *FOXE1* (*Forkhead factor E1*, also called *TTF2* for *Thyroid Transcription Factor 2*) and *ATM* (*Ataxia-Telangiectasia Mutated*) have been associated with DTC in other populations and are compelling candidates due to their roles in thyroid development or response to DNA damage, we chose to assess the contribution of genetic variations in or near these three genes to the risk of DTC in the Cuban population.

*NKX2-1* and *FOXE1* encode thyroid-specific transcription factors that play an important role in thyroid development and whose expression is modified in thyroid tumours [5-7]. The first thyroid cancer genome-wide association study (GWAS) reported the contribution of two SNPs near these two genes, namely rs944289, located 337-kb upstream of *NKX2-1* on chromosome 14q13.3, and rs965513, located 57-kb upstream of *FOXE1* on chromosome 9q22.33, to the risk of developing DTC in the European population [8]. Subsequently, the relationship between these two loci and DTC susceptibility has been investigated in other populations, but these associations vary in the context of different ethnic backgrounds and *FOXE1* polymorphisms were more strongly correlated with the pathogenesis of PTC than *NKX2-1* polymorphisms [9-12]. In particular, two functional polymorphisms in *FOXE1* appeared to be of specific interest: rs1867277, located within the 5′ untranslated region (UTR) and involved in the allele-specific transcriptional regulation of *FOXE1* through recruitment of the USF1/USF2 transcription factors [13-16], and rs71369530, the poly-alanine expansion in the *FOXE1* coding region [17,18].

ATM is a key initiator of the DNA damage response and some *ATM* SNPs have been reported to play a role in hormone dependent cancers and radiation sensitivity [19]. In particular, the common missense substitution D1853N (rs1801516) has been shown to play a role in DTC risk following irradiation [16,20] but the association with sporadic PTC was not replicated in a meta-analysis [11].

## Results

For each genotyped polymorphism, allele and genotype frequencies were calculated and Hardy-Weinberg equilibrium (HWE) was tested in the studied sample set. The five polymorphisms were in HWE among the analysed control subjects (Table 1). We noted that in the control population the minor allele frequency (MAF) of rs944289 near *NKTX2-1* was significantly lower in subjects of African origin than in the others (p = 0.03). No significant difference in MAF was observed for the other tested polymorphisms between the different ethnic groups (p < 0.3, whatever the polymorphism).

**Table 1:** **Description of the five studied polymorphisms**

**Reference**	**Location**	**Chromosome**	**Polymorphism**		**Minor allele frequency in cases**			**Minor allele frequency in controls**			**Hardy-Weinberg equilibrium** χ^**2**^ **p-value**	
			**Allele change**	**Residue change**	**All**	**African origin**	**Others**	**All**	**African origin**	**Others**	**Cases**	**Controls**
rs1801516	Coding region of *ATM*	11q22–23	G > A	D1853N	0.11	0.14	0.10	0.11	0.13	0.10	0.08	0.7
rs944289	Intergenic, 337 kb telomeric of *NKX2–1*	14q13.3	C > T	-	0.56	0.46	0.59	0.43	0.36	0.47	0.04	0.5
rs965513	Intergenic, 57 kb upstream to *FOXE1*	9q22.33	G > A	-	0.36	0.28	0.39	0.25	0.21	0.26	0.6	0.9
rs1867277	5′UTR of *FOXE1*	9q22.33	G > A	-	0.46	0.40	0.48	0.37	0.38	0.36	0.7	0.4
rs71369530	Exon 1 of *FOXE1*	9q22.33	-	Poly-alanine tract expansion	0.40	0.29	0.43	0.28	0.29	0.27	0.6	0.9

When stratifying by sex and age, and adjusting for body surface area (BSA), body mass index (BMI), size, ethnicity, tobacco consumption and, for women, number of pregnancies, all tested SNPs but rs1801516 in *ATM* (D1853N) were found to be associated with increased risk of DTC in the Cuban population (Table 2). ORs per minor allele, ranged from 1.5 (95% CI: 1.1–1.9) for rs1867277 located in the 5′UTR of *FOXE1* to 1.8 (95% CI: 1.3–2.5) for the length polymorphism rs71369530 in the coding sequence of *FOXE1* (Table 2).

**Table 2:** **Association results between the five polymorphisms and the risk of developing DTC**

**Genotypes**	**Genotyped participants**		**Crude OR** ^**a**^ **(95% CI)**	**p-value**	**Adjusted OR** ^**b**^ **(95% CI)**	**Adjusted OR per allele (95% CI)**	**p-value**
	**Cases n (%)**	**Controls n (%)**					
rs1801516 (*ATM*)	n = 197	n = 206					
G/G	153	162	Ref		Ref		
G/A	44	42	1.2 (0.7–1.9)	0.6	1.2 (0.7–2.0)	1.1 (0.7–1.7)	0.8
A/A	0	2	-	0.9	-		
rs944289 (near *NKX2-1*)	n = 202	n = 209					
C/C	47	70	Ref		Ref		
C/T	85	98	1.3 (0.8–2.0)	0.4	1.2 (0.7–1.9)	1.6 (1.1–2.1)	0.02
T/T	70	41	2.6 (1.5–4.5)	<0.001	2.5 (1.4–4.4)		
rs965513 (near *FOXE1*)	n = 203	n = 212					
G/G	83	118	Ref		Ref		
G/A	91	81	2.0 (1.0–4.1)	0.06	1.7 (1.1–2.6)	1.7 (1.2–2.3)	0.02
A/A	29	13	3.3 (1.16–6.9)	0.001	2.9 (1.3–6.1)		
rs1867277 (5′UTR of *FOXE1*)	n = 203	n = 212					
G/G	56	88	Ref		Ref		
G/A	104	91	1.1 (0.7–1.9)	0.6	1.8 (1.2–2.9)	1.5 (1.1–1.9)	0.01
A/A	43	33	2.1 (1.2–3.8)	<0.01	1.9 (1.1–3.5)		
rs71369530 (length polymorphism in *FOXE1*)	n = 203	n = 212					
S/S^c^	72	112	Ref		Ref		
S/L^c^	101	84	1.5 (0.8–3.0)	0.2	2.0 (1.3–3.1)	1.8 (1.3–2.5)	0.0003
L/L^c^	30	16	2.9 (1.5–5.7)	<0.005	2.9 (1.4–5.9)		

^a^Stratified by age and sex.

^b^Stratified by age and sex and adjusted on BSA, ethnicity, tobacco, size and for women, number of pregnancies.

^c^S for alleles with 14 or fewer alanine residues and L for alleles with 16 or more alanine residues.

A systematic investigation of potential interactions between the five polymorphisms and ethnicity, BMI, BSA, size, tobacco consumption and, for women, parity (Tables 3 and 4), revealed a suggestive interaction (p = 0.03) between the *ATM* coding SNP and the number of pregnancies. Women who had 2 or more pregnancies had a 3.5-fold (95% CI: 1.2–9.8) increase in risk of DTC if carrying the A allele whereas this OR was 0.8 (95% CI: 0.4–1.6) in women having had none or one pregnancy only (Table 3).

**Table 3:** **Results of interaction tests between genetic factors and other putative risk factors for DTC**

		**rs944289** ^**$**^ **(near** ***NKX2-1*** **)**			**p-interaction**	**rs1801516** ^**$**^ **(** ***ATM*** **)**			**p- interaction**
		**C/C**	**C/T**	**T/T**		**G/G**	**G/A**	**A/A**	
Ethnicity									
African	Cases/controls	16/33	21/28	12/12		35/54	14/18	0/0	
	OR (95% CI)	Ref	1.5 (0.7–3.5)	2.3 (0.8–6.3)	0.9	Ref	1.2 (0.6–2.7)		
Other	Cases/controls	31/37	64/70	58/29		118/108	30/24	0/2	0.9
	OR (95% CI)	Ref	1.1 (0.6–2.0)	2.4 (1.2–4.5)		Ref	1.2 (0.7–2.2)	-	
BMI (kg/m^2^)									
≤ Median	Cases/controls	27/40	33/53	19/22		68/90	21/21	0/2	
	OR (95% CI)	Ref	0.9 (0.4–1.8)	2.1 (1.0–4.4)	0.4	Ref	1.3 (0.6–2.4)	_	0.5
> Median	Cases/controls	20/30	52/45	40/19		85/72	23/21	0/0	
	OR (95% CI)		1.7 (0.8–3.4)	3.1 (1.4–6.8)		Ref	1.0 (0.5–1.9)	-	
BSA (m^2^)									
≤ Median	Cases/controls	27/41	32/52	34/24		72/93	20/22	0/1	
	OR (95% CI)	Ref	1.0 (0.5–1.9)	2.2 (1.0–4.4)	0.5	Ref	1.2 (0.6–2.4)	_	0.9
> Median	Cases/controls	20/29	53/46	36/17		81/69	24/20	0/1	
	OR (95% CI)	Ref	1.6 (0.6–3.3)	3.1 (1.4–7.0)		Ref	1.0 (0.5–2.0)	-	
Size (m)									
≤ Median	Cases/controls	26/39	43/47	36/21		79/84	23/22	0/0	
	OR (95% CI)	Ref	1.3 (0.7–2.6)	2.6 (1.2–5.4)	0.9	Ref	1.2 (0.6–2.3)	-	
> Median	Cases/controls	21/31	42/51	34/20		74/78	21/20	0.2	0.8
	OR (95% CI)	Ref	1.3 (0.6–2.5)	2.6 (1.2–57)		Ref	1.0 (0.5–2.1)	-	
Ever smoker									
No	Cases/controls	33/46	56/56	54/22		107/98	32/22	0/2	
	OR (95% CI)	Ref	1.2 (0.5–2.7)	1.4 (0.6–3.6)		Ref	1.4 (0.8–2.7)	-	0.2
Yes	Cases/controls	14/24	29/42	16/19	0.1	46/64	12/20	0	
	OR (95% CI)	Ref	1.4 (0.8–2.5)	3.5 (1.8–7.1)		Ref	0.8 (0.3–0.7)	-	
Pregnancy									
<2	Cases/controls	19/39	35/54	35/26		72/91	17/25	0/2	
	OR (95% CI)	Ref	1.3 (0.7–2.6)	2.7 (1.2–5.7)		Ref	0.8 (0.4–1.6)	-	
≥2	Cases/controls	24/21	38/22	27/9	0.9	61/46	23/5	0/0	0.03
	OR (95% CI)	Ref	1.5 (0.7–3.4)	2.6 (1.0–6.8)		Ref	3.5 (1.2–9.8)	-	

All ORs and tests are stratified on sex and age.

$ Not including missing data.

**Table 4:** **Results of interaction tests between genetic factors and other putative risk factors for DTC**

		**rs965513** ^**$**^ **(near** ***FOXE1*** **)**			**P-interaction**	**rs1867277** ^**$**^ **(5′UTR of** ***FOXE1*** **)**			**p-interaction**	**rs71369530** ^**$**^ **(length polymorphism in** ***FOXE1*** **)**			**p-interaction**
		**G/G**	**G/A**	**A/A**		**G/G**	**G/A**	**A/A**		S/S*	L/S*	L/L*	
Ethnicity													
African	Cases/controls	25/41	18/28	4/1		18/30	23/32	8/12		24/37	22/31	3/6	
	OR (95% CI)	Ref	1.1 (0.5–2.4)	6.4 (0.7–0.4)	0.3	Ref	1.2 (0.5–2.7)	1.1 (0.4–3.1)	0.4	Ref	1.1 (0.5–2.4)	0.8 (0.2–3.3)	0.09
Other	Cases/controls	56/75	71/49	23/11		38/57	81/59	33/20		48/73	79/52	27/10	
	OR (95% CI)	Ref	2.0 (1.2–3.2)	2.9 (1.3–6.4)		Ref	2.0 (1.2–3.5)	2.4 (1.2–4.9)		Ref	1.1 (0.5–2.3)	4.0 (1.8–3.2)	
BMI (kg/m^2^)													
≤ Median	Cases/controls	36/63	43/40	10/8		25/52	46/48	19/15		32/61	45/44	13/9	
	OR (95% CI)	Ref	1.9 (1.0–3.4)	2.0 (0.7–5.6)	0.3	Ref	2.0 (1.1–3.7)	1.6 (1.1–6.0)	0.6	Ref	1.9 (1.1–3.5)	2.7 (1.1–6.9)	0.9
> Median	Cases/controls	45/53	46/37	17/4		31/35	58/43	22/17		40/49	56/39	17/7	
	OR (95% CI)	Ref	1.5 (0.8–2.8)	5.7 (1.8–8.6)		Ref	1.6 (0.8–3.0)	1.4 (0.6–3.2)		Ref	1.8 (1.0–3.2)	2.9 (1.1–7.6)	
BSA (m^2^)													
≤ Median	Cases/controls	37/63	41/44	13/8		27/49	49/53	17/16		35/62	44/46	15/10	
	OR (95% CI)	Ref	1.6 (0.9–2.9)	2.8 (1.1–7.5)	0.8	Ref	1.6 (0.9–3.0)	1.9 (0.8–4.4)		Ref	1.7 (0.9–3.0)	2.6 (1.1–6.5)	0.9
> Median	Cases/controls	44/53	48/33	14/4		29/38	55/38	24/16	0.9	37/48	57/47	15/6	
	OR (95% CI)	Ref	1.8 (1.0–3.4)	4.4 (1.3–14.6)		Ref	2.0 (1.0–3.8)	1.9 (0.9–4.3)		Ref	2.1 (1.1–3.8)	3.1 (1.1–8.7)	
Size (m)													
≤ Median	Cases/controls	42/59	42/41	16/6		28/48	58/44	20/16		35/55	55/42	16/11	
	OR (95% CI)	Ref	1.4 (0.8–2.7)	3.8 (1.4–10.6)	0.7	Ref	2.5 (1.4–4.7)	2.3 (1.0–5.2)	0.3	Ref	2.0 (1.1–3.6)	2.2 (0.9–5.3)	0.5
> Median	Cases/controls	39/57	47/36	11/6		30/39	46/57	21/16		37/55	46/41	14/5	
	OR (95% CI)	Ref	2.0 (1.1–3.6)	2.8 (0.9–8.3)		Ref	1.3 (0.7–2.4)	1.7 (0.8_3.8)		Ref	1.7 (0.9–3.1)	4.2 (1.4–12.5)	
Ever smoker													
No	Cases/controls	57/70	60/46	22/6		43/55	71/54	28/16		55/68	68/49	21/6	
	OR (95% CI)	Ref	1.7 (1.0–2.8)	4.8 (1.8–12.7)		Ref	1.7 (1.0–2.9)	2.1 (1.0–4.4)	0.3	Ref	1.7 (1.0–2.8)	4.2 (1.6–11.1)	0.3
Yes	Cases/controls	24/46	29/31	5/6	0.3	13/32	33/37	13/16		17/42	33/34	9/10	
	OR (95% CI)	Ref	1.8 (0.9–3.7)	1.4 (0.4–5.0)		Ref	2.2 (1.0–4.9)	1.9 (0.7–5.1)		Ref	2.4 (1.1–5.0)	2.1 (0.7–6.1)	
Pregnancies													
<2	Cases/controls	34/68	44/42	10/6		23/55	50/48	16/17		31/63	46/48	12/8	
	OR (95% CI)	Ref	2.0 (1.1–3.7)	3.1 (1.0–9.2)	0.8	Ref	2.5 (1.3–4.6)	2.2 (0.9–5.0)		Ref	2.0 (1.1–3.6)	2.9 (1.1–7.9)	
≥2	Cases/controls	38/30	36/18	12/3		25/15	42/30	21/7	0.4	33/27	41/21	16/3	0.7
	OR (95% CI)	Ref	1.6 (0.8–3.2)	3.1 (0.8–12.1)		Ref	0.8 (0.4–1.8)	1.7 (0.6–5.1)		Ref	1.6 (0.8–3.3)	4.3 (1.1–16.5)	

All ORs and tests are stratified on sex and age.

*S for Short alleles (12–14 alanines) and L for Long allele (16–19 alanines).

^$^Not including missing data.

## Discussion

In the present work, we assessed the relationship between five putative or recognized polymorphisms involved in DTC risk in the Cuban population, where the incidence of thyroid cancer is particularly low [4].

We replicated the association between polymorphisms at *NKX2-1* and *FOXE1* loci and DTC risk previously reported in a GWAS in European [8] and Japanese [14] populations. When taking into account multiple tests using Bonferoni correction [19], significant threshold for p-value should be 0.01, and only 2 of the 3 tested SNPs at *FOXE1* loci remained significant.

The power of the present study was low for the *ATM* SNP rs1801516, because of the low MAF (11%) in controls. For this SNP, a power of 80% is reached only for an OR of 3.5 or higher. For the other tested SNPs with MAFs of about 20% in controls (range: 15% to 27%, Table 1), our study had a power of 80% for evidencing an association if OR is 1.7 or higher, when not taking into account multiple tests, and if OR is 2.0 or higher when taking in account multiple testing. Only important interaction could be evidenced given the size of our study, a power of 80% being reached for gene-environment interactions of a factor 3, assuming an environmental factor present in 50% of controls, a main OR for environmental factor equal to 2, a SNP MAF equal to 20% and a main OR per minor allele equal to 1.5. All these numbers being given without correction for multiple tests.

In addition to a low power, our study suffers from the traditional limitations of case-control studies. If size, pregnancies number and smoking habits are probably well reported by subjects, it is impossible for us to verify that cases correctly reported their weight before thyroid cancer, as specified in questionnaire, rather than their weight at time of interview.

Although we did not evidence an association between *ATM* D1853N (rs1801516) and DTC risk in the whole study set, a significant (p = 0.03) interaction was found in women between this polymorphism and the number of pregnancies, which is another known risk factor for DTC [4]. In the Cuban study the minor allele (A) was significantly associated with a 3-fold increased risk of DTC among women who had had two or more children.

Interestingly, as observed in the Cuban population [4], an increased risk of DTC with increasing number of pregnancies had been observed in natives of French Polynesia (OR = 3.1, 95% CI: 1.2–8.3) [21], where the average number of children is very high (about 4 children per woman in the controls). In the French Polynesian study the minor allele *A* was quite rare in the population (2% in controls), but it was associated with a significantly increased risk of DTC (Maillard et al. submitted).

These observations raise interesting questions about the biological role of *ATM*, and possibly of other DNA repair genes, on the development of hormone-related cancers. The Ser/Thr protein kinase *ATM* is primarily known as a central element of the cellular response to double-strand break (DSB) lesions. DNA DSBs can be generated by DNA damaging agents, such as ionizing radiation, following the collapse of stalled replication forks or the response to uncapped telomeres. Unrepaired DSBs can severely disrupt DNA replication in proliferating cells, usually leading to cell death, or leave chromosomal aberrations leading to cancer formation. In previous studies on radio-induced PTC or sporadic PTC, the missense substitution D1853N in *ATM* had been associated with a decreased risk [16,22]. More recently, it has been reported that this conserved variant falling just upstream of the FAT kinase domain [23] may modify the genetic susceptibility to DTC and its clinical manifestation in carriers of a rare BRCA1 pathogenic variant. In particular, both *ATM* rs1801516 and *BRCA1* rs16941 variants modify the impact of male gender on clinical variables [24]. An emerging hypothesis is that ATM is exploited in undamaged cells in other signalling pathways that DSBs repair in response to various stimuli or physiological situations such as hormonal exposure [25]. One could also hypothesize that oestrogen could contribute to DTC via the induction of DNA damage. For instance, in breast cancer it has been proposed that oestrogen receptor signalling converges to suppress effective DNA repair and apoptosis in favour of proliferation [26]. A variation in breast cancer risk associated with parity has been evidenced according to the type of mutation in the DNA repair gene *BRCA1*, acting in the same pathway as *ATM* [27]. Hence, further studies are warranted to better understand the role of ATM in hormone-related cancers such as DTC.

## Conclusions

We confirmed in the Cuban population the role of the loci that have been previously associated with DTC susceptibility in European and Japanese populations through genomewide association studies. Moreover, our result on *ATM* and the number of pregnancies raises interesting questions on the mechanisms by which oestrogens, or other hormones, alter the DNA damage response and DNA repair through the regulation of key effector proteins such as ATM. Due to the small size of our study and to multiple testing, all these results warrant further investigation in a larger sample set.

## Methods

This case-control study was carried out in Havana, Cuba, and was revised and approved by the Clinical Research Ethics Committee of the National Institute of Oncology (INOR), Havana, Cuba. Informed written consent was obtained from all study participants.

### Subjects selection and interviews

The cases and controls selection process as well as the case-control study methodology have been described elsewhere [4]. In brief, cases lived in the Havana area, were between 18 and 50 years old at time of DTC diagnosis and had been treated between 2000 and 2011 at INOR or at the Institute of Endocrinology of Havana. Of the 240 eligible DTC cases, 37 (15%) individuals were not interviewed because they could not be located (n = 32) or refused to participate (n = 5). The final study population consisted of 203 cases. Controls were selected from the general population living in the same areas using consultation files from primary care units (family doctors). They were frequency-matched with cases by age at cancer diagnosis (±5 years) and gender. Of the 229 potential controls, 17 refused and 212 agreed to be interviewed.

All 415 participants were interviewed face-to-face by trained professionals (nurses and medical staff) using a structured questionnaire between January 2009 and December 2011 in presence of a parent, a relative, or a general practitioner. Cases and controls characteristics are described in Table 5. All participants gave their consent for saliva sampling and genetic analyses.

**Table 5:** **Characteristics of the 415 subjects participating in the case-control study**

**Characteristics**	**Controls (n = 212) (%)**	**Cases (n = 203) (%)**
Gender		
Male	39 (18.4)	24 (11.8)
Female	173 (81.6)	179 (88.2)
Age at diagnosis (years)		
<25	24 (11.3)	23 (11.3)
25–34	47 (22.2)	40 (19.7)
35–44	87 (41.0)	87 (42.9)
45–54	49 (23.1)	46 (22.7)
55+	5 (2.4)	7 (3.4)
Histology		
Papillary thyroid carcinoma	*N/A*	162 (89.5)
Follicular thyroid carcinoma	*N/A*	19 (10.5)
Missing	*N/A*	22
Ethnicity		
European	38 (17.9)	57 (28.1)
Mixed	99 (46.7)	97 (47.8)
African	75 (35.4)	49 (24.1)
Body Mass Index (kg/m^2^)		
> Median in genotyped controls	105 (49.5)	79 (38.9)
≤ Median in genotyped controls	107 (50.5)	124 (61.1)
Body surface area (m^2^)		
> Median in genotyped controls	105 (49.5)	85 (41.9)
≤ Median in genotyped controls	107 (50.5)	118 (58.1)
Smoker		
Never smoker	125 (59.0)	144 (70.9)
Current or former smoker	87 (41.0)	59 (29.1)

N/A: not applicable.

### DNA isolation

Saliva samples were collected using a DNA Genotek Oragene DNA collection kit (Ottawa, Canada). Genomic DNA (gDNA) was extracted using a standard inorganic method (Qiagen Autopure LS, Courtaboeuf, France). The gDNA was then quantified with the Life Technologies Picogreen kit (Saint-Aubin, France). For the genotyping, DNA from study participants was randomized on plates and all samples were analysed simultaneously. For quality control purposes, duplicates of 10% of the samples were interspersed throughout the plates.

### Genotyping

Five polymorphisms that were observed in previous studies to be associated with DTC were selected for genotyping: the nonsynonymous SNP rs1801516 (D1853N) in *ATM*, the GWAS SNP rs944289 near *PTCSC3* and *NKX2-1* at 14q13.3, the GWAS SNP rs965513 near *FOXE1* at 9q22.33, rs1867277 in the 5′UTR of *FOXE1*, and the poly-alanine stretch polymorphism rs71369530 in *FOXE1* that is the result of a variable number of alanine repeats.

For SNPs rs944289, rs965513, rs1867277, and rs1801516, 25 ng of gDNA were analysed using High-Resolution Melting curve (HRM) with a specific probe. Some representative samples were re-sequenced by dye-terminator to confirm the genotype [28]. Fluorescence readings and data analyses were done with the Idaho Technology LightScanner Inc. Hi-Res Melting System (Idaho Technology, Salt Lake City, UT).

For rs71369530, 30 ng of gDNA was amplified by PCR with fluorescently end-labelled forward primers (5′–6-FAM or 5′-HEX) using KAPA 2G Fast HotStart ReadyMix (KAPA Biosystems, Woburn, MA, US) in a 10 μl final reaction volume (0.5 mM MgCl2, 5% DMSO, 0.25 mM primers). The fluorescently-labelled PCR product was loaded on an ABI 3730 capillary sequencer and analysed as a variable length fragment polymorphism using GenScan size standards (ROX–500) as internal size standards. Data were collected and visualized with Genotyper Software v3.7. To determine the number of repeats corresponding to each allele identified in the genotyping assay, the PCR products from 6 homozygous individuals were Sanger sequenced.

The sequences of all PCR primers, HRM probes, and all PCR conditions are available from the authors on request.

The proportion of successfully genotyped DNA samples was 99.0% for rs944289, 96.9% for rs965513, 99.0% for rs1867277, 99.3% rs71369530, and 97.1% for rs1801516. Quality control analysis showed a concordance rate >99% between duplicate samples.

### Statistical analyses

For the statistical analyses, the study participants were classified into three categories according to the ethnicity of their parents: European (both parents of European origin), African (both parents of African origin), and other (all other combinations of parental origin). Body mass index (BMI) was defined as weight (kg) divided by height (m) squared, and body surface area (BSA) was calculated using the Boyd formula: BSA(m^2^) = 0.0003207 × (weight)^0.7285 – (0.0188 * log (weight))^ × (height)^0.3^, where weight is expressed in g and height in cm [29]. Quantitative factors were categorised into tertiles based on their distribution among the controls. Anthropometric categorisation was defined separately for men and for women.

Allele and genotype frequencies were calculated and HWE was tested using a χ^2^ test in the studied sample set for each polymorphism. The five SNPs were in HWE among the analysed control subjects (Table 1).

For the genotype analysis of the *FOXE1* multi-allelic poly-alanine stretch length polymorphism (rs71369530), we considered a bi-allelic marker with the three possible genotypes according to the length of the alanine tract: Short/Short, Short/Long and Long/Long, with short alleles (S) including alleles coding for a stretch of 12–14 alanines and long alleles (L) comprising those alleles coding for a stretch of 16–19 alanines.

Nineteen strata were defined based on age and gender, seven for men and twelve for women. The association between the five analysed polymorphisms and risk of DTC was assessed using multiple logistic regressions and assuming co-dominant, dominant, and recessive genetic models of inheritance [30,31]. Crude analyses and analyses adjusted for environmental thyroid cancer risk factors were performed. Tests for interaction were performed to determine whether the putative associations of SNPs with the risk of developing DTC were modified by environmental parameters [30]. All statistical analyses were done with SAS software, version 9.3 (SAS Institute Inc, NC, USA).

## Abbreviations

DTC: Differentiated thyroid carcinoma
SNP: Single nucleotide polymorphism
GWAS: Genome-wide association study
PTC: Papillary thyroid carcinoma
HWE: Hardy-Weinberg equilibrium
MAF: Minor allele frequency
BSA: Body surface area
BMI: Body mass index
DSB: Double-strand break
HRM: High-resolution melting curve
OR: Odds-ratio
